# H3K27me3-mediated epigenetic regulation in pluripotency maintenance and lineage differentiation

**DOI:** 10.1016/j.cellin.2024.100180

**Published:** 2024-06-27

**Authors:** Liwen Jiang, Linfeng Huang, Wei Jiang

**Affiliations:** a Department of Biological Repositories, Frontier Science Center for Immunology and Metabolism, Medical Research Institute, Zhongnan Hospital of Wuhan University, Wuhan University, Wuhan, 430071, China; b Wang-Cai Biochemistry Lab, Division of Natural and Applied Sciences, Duke Kunshan University, Kunshan, Jiangsu, China; c Hubei Provincial Key Laboratory of Developmentally Originated Disease, Wuhan, 430071, China

**Keywords:** H3K27me3, PRC2, KDM6, Pluripotency, Lineage differentiation

## Abstract

Cell fate determination is an intricate process which is orchestrated by multiple regulatory layers including signal pathways, transcriptional factors, epigenetic modifications, and metabolic rewiring. Among the sophisticated epigenetic modulations, the repressive mark H3K27me3, deposited by PRC2 (polycomb repressive complex 2) and removed by demethylase KDM6, plays a pivotal role in mediating the cellular identity transition through its dynamic and precise alterations. Herein, we overview and discuss how H3K27me3 and its modifiers regulate pluripotency maintenance and early lineage differentiation. We primarily highlight the following four aspects: 1) the two subcomplexes PRC2.1 and PRC2.2 and the distribution of genomic H3K27 methylation; 2) PRC2 as a critical regulator in pluripotency maintenance and exit; 3) the emerging role of the eraser KDM6 in early differentiation; 4) newly identified additional factors influencing H3K27me3. We present a comprehensive insight into the molecular principles of the dynamic regulation of H3K27me3, as well as how this epigenetic mark participates in pluripotent stem cell-centered cell fate determination.

Pluripotent stem cells (PSCs) mainly include embryonic stem cells (ESCs) and induced pluripotent stem cells (iPSCs). ESCs are derived from the inner cell mass of blastocyst, whereas iPSCs are produced by overexpressing specific transcriptional factors or treatment with chemical compounds (Wang et al., 2023; Young, 2011). Both PSCs have the abilities of self-renewal and differentiation into any embryonic lineages. Therefore, PSCs enable a wide range of applications in generating functional cells, investigating the mechanisms of early development as well as drug screening (Wang et al., 2023; Young, 2011). In the processes of PSC self-renewal and differentiation, epigenetic regulation safeguards cell identities through establishing distinctive epigenetic landscapes (Gokbuget & Blelloch, 2019). The bivalent domains, which comprise both the repressive epigenetic mark H3K27me3 and the active epigenetic mark H3K4me3, distinctively occupy genes associated with lineage specification in ESCs (Bernstein et al., 2006). Bivalent domains are thus considered as poised marks normally repressing the aberrant activation of lineage-specific genes while yet simultaneously possessing the potential to initiate differentiation (Bernstein et al., 2006). PRC2 is a large complex depositing repressive H3K27me3 in bivalent domains and its role in condensing the chromatin and further limiting the access of transcriptional factors has been widely adopted (Blackledge & Klose, 2021). In this review we emphasize how the bivalent domain-associated PRC2 precisely regulates lineage commitment and its underlying mechanisms based on recent literature; moreover, we also discuss additional factors that influence the H3K27me3 and cell fate determination.

## 1 PRC2.1/2.2 and their genome-wide distributions

**Fig. 1:**
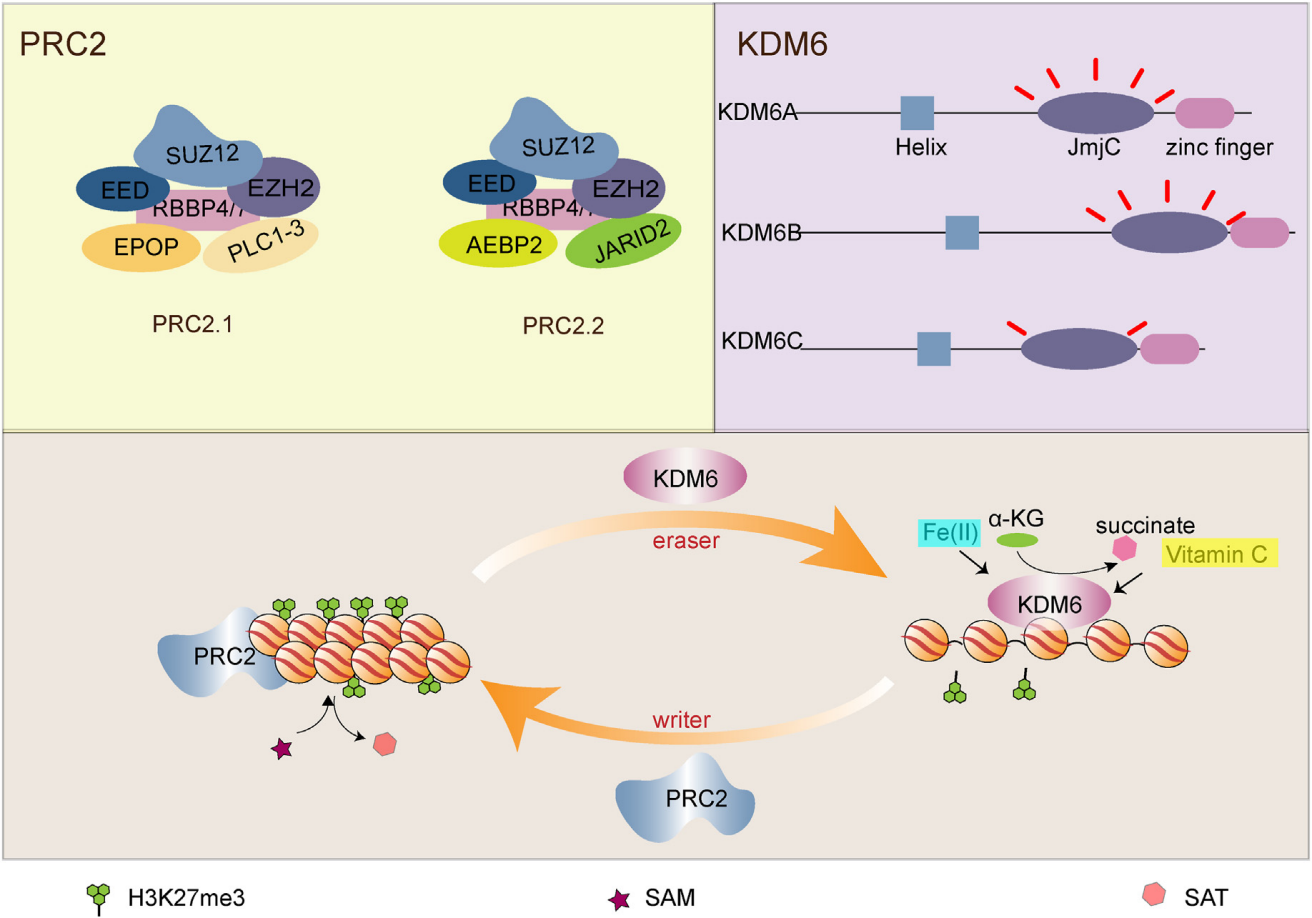
**The methylation and demethylation of H3K27me3.** PRC2 is composed of core subunits (EZH1/EZH2, EED, SUZ12, and RBBP4/RBBP7), and the accessory proteins which define the subcomplex: PRC2.1 contains PCL1/2/3 and EPOP while PRC2.2 has JARID2 and AEBP2. KDM6 family contains three members KDM6A/KDM6B/KDM6C while KDM6C exhibits very weak catalytic activity. Functionally, PRC2 establishes the H3K27me3 pattern on the bivalent developmental genes to diminish chromatin accessibility whereas KDM6 loosens the chromatin structure and contributes to the bivalency resolution.

PRC2 is composed of four core subunits (Fig. 1): EZH1 (enhancer of zeste homologue 1) or its paralog EZH2, EED (embryonic ectoderm development), SUZ12 (suppressor of zeste 12), and RBBP4 (RB binding protein 4) or RBBP7 (Blackledge & Klose, 2021; Laugesen et al., 2019). The major subunit H3K27 methyltransferase EZH1/2 deposits H3K27me2/me3 or H3K27me1, although the role of PRC2 in depositing H3K27me1 is still controversial (Margueron & Reinberg, 2011; Piunti & Shilatifard, 2021). To get insight into the distribution of PRC2 across mouse and human genomes, van Steensel and colleagues performed a genome-wide chromatin immunoprecipitation followed by sequencing (ChIP-seq) using antibodies against the core components EZH2 and SUZ12 and found most of the PRC2 binding sites located at the promoters of developmental genes (van Steensel et al., 2008), a pattern very similar to the genomic distribution of H3K27me3 (Pan et al., 2007; van Steensel et al., 2008). In mouse ESCs, H3K27me2 localizes at nearly 50%–70% of H3 as the predominant type of H3K27 methylation while H3K27me1 and H3K27me3 localize at only 10%–15% of H3. Furthermore, these different H3K27 methylation forms have distinctive distribution features and functions. H3K27me3 is often established on CpG islands (CGIs) of promoters to repress genes and H3K27me2 occupies the intergenic or intragenic regions with potential roles in preventing abnormal activation of non-cell type specific enhancers or promoters. On the contrary, H3K27me1 contributes to gene activation through the H3K36me3-dependent way (Ferrari et al., 2014; Højfeldt et al., 2018). Such differences shed light on the complicated mechanisms mediated by PRC2 and H3K27 methylation in regulating gene expression and cell fate determination. H3K27me3 is primarily located in transcription start sites of development-related genes such as the *HOX*, *FOX*, *SOX*, and *TBX* gene families in both mouse and human ESCs; these genes are engaged in morphogenesis, pattern formation and embryonic development (Boyer et al., 2006; Lee et al., 2006). Intriguingly, some pluripotent transcriptional factors such as OCT4, SOX2 and NANOG co-occupy a large subset of these genes (Lee et al., 2006), suggesting the synergistic roles of H3K27me3 and pluripotent factors in pluripotency maintenance.

In addition to the core subunits, the accessory proteins define PRC2 into PRC2.1 and PRC2.2. PRC2.1 contains one of the three polycomb-like proteins (PCL1, 2, 3) and EPOP (elongin BC and polycomb repressive complex 2 associated protein) while PRC2.2 has the exclusive elements JARID2 (jumonji and AT-rich interaction domain containing 2) and AEBP2 (adipocyte enhancer-binding protein 2) (Healy et al., 2019; Højfeldt et al., 2019). Accumulating studies have reported the existence of PRC2 subcomplexes, but the functional relevance remains obscure. Notably, although PRC2.1 and PRC2.2 exhibit large overlaps at polycomb target sites, they indeed have varied chromatin binding affinities. In human stem cells exclusively expressing PRC2.1, SUZ12 shows increased occupancy specifically at polycomb target genes such as *PRDM12*, however, the signal of SUZ12 ChIP-seq reduces at *PRDM12* and other 307 genes in cells only expressing PRC2.2, suggesting a higher DNA binding affinity of PRC2.1 (Youmans et al., 2021). Ngan and colleagues analyzed the long-range chromatin interactions mediated by PRC2 using chromatin interaction analysis by paired-end tag sequencing (ChIA-PET). They found most of the PRC2-bound regions formed specific loops (Ngan et al., 2020), suggesting PRC2 could repress these developmental genes through chromatin loops, like how enhancers activate genes. It is noteworthy that PRC2-bound regions are often simultaneously enriched in poised enhancer marks H3K4me1 and H3K4me3, which indicates these silencers might be transformed into active enhancers during differentiation (Ngan et al., 2020).

## 2 PRC2 participates in pluripotency maintenance and early differentiation

**Fig. 2:**
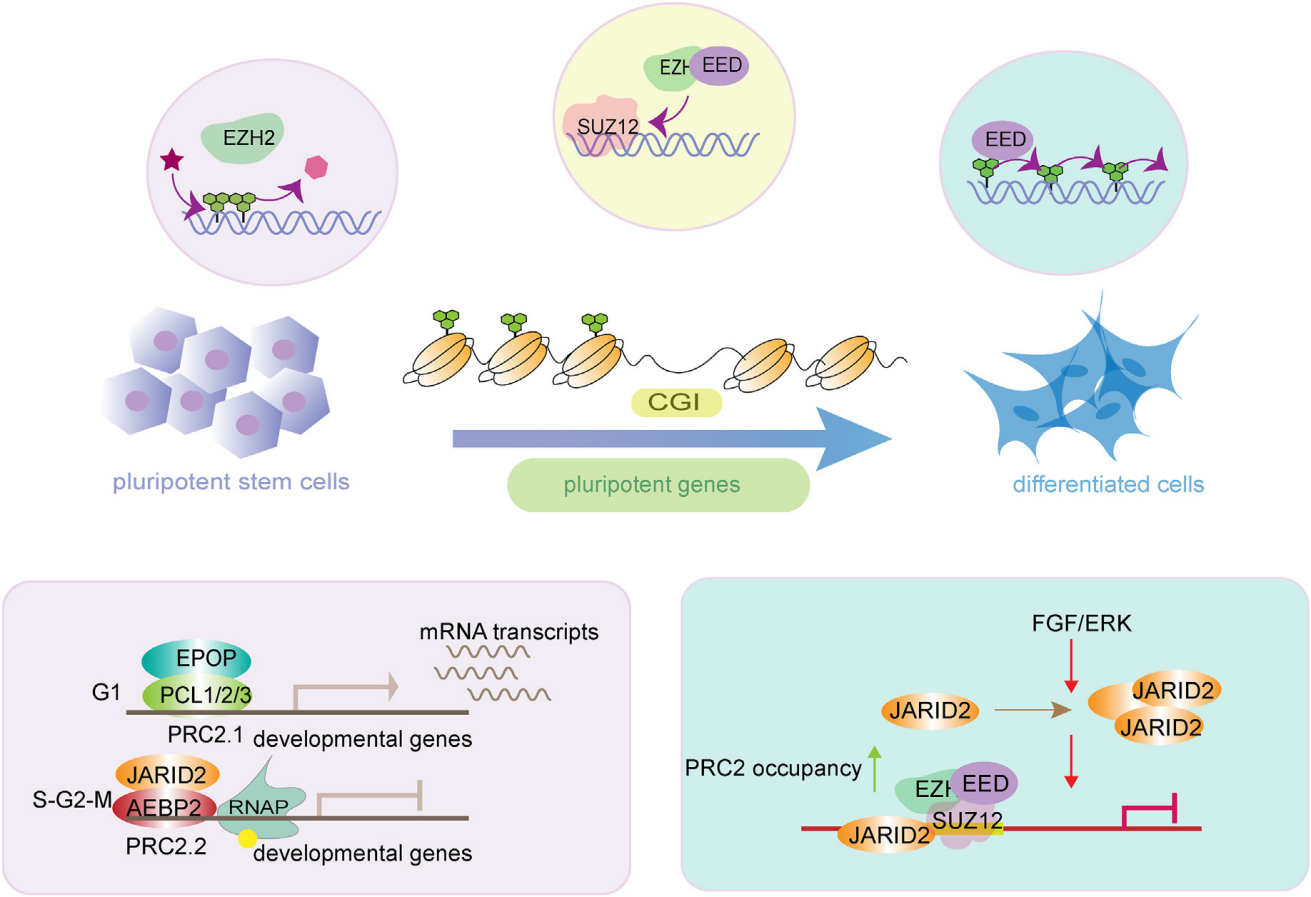
**PRC2 mediates cell identity transition between pluripotent and differentiated status.** The core subunits contribute to the establishment and maintenance of H3K27me3: EZH2 utilizes SAM as the metabolic substrate for histone methylation; SUZ12 promotes the EED-EZH2 complex formation and the recruitment of PRC2 compositions; EED accelerates the H3K27me3 propagation. The exclusive subunits of PRC2.1 and PRC2.2 involve in the cell cycle transition of mouse ESCs: JARID2-PRC2.2 binds to the bivalent promoter region and cooperates with the phosphorylated RNAPII to inhibit cell entry into the S and G2 phases and EPOP of PRC2.1 acts as a transcriptional activator that binds to the promoter region in the G1 phase. In addition, the FGF/ERK signal pathway could upregulate JARID2 expression, which subsequently influences the PRC2 occupancy to safeguard the transcriptional poised state.

The polycomb repressive system, which comprises two large complexes PRC1 and PRC2, plays an important role in development. The two complexes share a large overlap at target regions on the genome. PRC2 deposits H3K27me3 at target sites while PRC1 further binds to the same region and deposits H2AK119ub. Both marks make the chromatin inaccessible, and thus limit the transcription of developmental regulators (Bracken et al., 2006; Fursova et al., 2019; Gao et al., 2012). To summarize, the overall function of PRC2 can be regulated by its subunits or by some newly discovered assembly factors. The absence of *SUZ12* prevents PRC2 from assembling and erases H3K27me3 (Højfeldt et al., 2018). The *Eed* deletion disrupts PRC2 and reduces the binding of PRC2 at the target genes such as *Gata4* and *Gata6* in mouse ESCs (Boyer et al., 2006). Interestingly, the JAZF1-SUZ12 fusion protein affects the assembly of PRC2 complex and generally reduces the binding of PRC2 to target sites on chromatin (Ma et al., 2017). The methylation of JARID2, mediated by PRC2 as well, can reciprocally promote the PRC2 enzymatic activity and safeguard the H3K27me3 occupation during cell differentiation (Sanulli et al., 2015). A recent report has shown that the alternative splicing product SUZ12-s of SUZ12 can mediate the dimerization of PRC2 and promote the deposition of H3K27me3 at the promoters of PRC2 target genes (Arecco et al., 2024). All these results indicate the complexity of the PRC2 regulatory model. Since PRC2 is the key repressor complex that deposits H3K27me3, how each component finely modulates H3K27me3 and thus enables early fate regulation has been extensively explored. In the following section, we will summarize how the individual subunits of PRC2 precisely establish, expand, and balance the role of H3K27me3 in pluripotency maintenance and early lineage differentiation (Fig. 2).

### 2.1 EZH2 and EZH1: exert the enzymatic activity

As the core catalytic subunit in the PRC2 complex, EZH1/2 is involved in cell fate determination of PSCs by catalyzing the H3K27me3 of either lineage-specific or pluripotency-associated transcriptional factors. Although there are two paralogs (EZH1 and EZH2), the PRC2-EZH1 and PRC2-EZH2 can mediate transcriptional repression through distinct mechanisms. PRC2-EZH2 represses genes mainly by establishing H3K27me2/3, whereas PRC2-EZH1 could participate in chromatin condensation of target genes irrespective of methyltransferase activity, which is not accompanied by changes of H3K27me2/me3 levels (Margueron et al., 2008). Nevertheless, activation of the PRC2 complex is a critical step to repress the genes. Intriguingly, distinct activation mechanisms of enzymatic activity are utilized for the two paralogs. Only EZH2 can be activated by allosteric regulators and is more sensitive to inhibitors of the methylation substrate SAM (Lee et al., 2018). The different modes of activation of EZH2/EZH1 illustrate the evolution of PRC2, which shares the function of EZH and tightly regulates gene repression.

In mouse ESCs, EZH2 is dispensable for pluripotency due to the compensatory role of its paralog EZH1. EZH1 can preserve the H3K27me3 target genes related to lineage development and safeguard the pluripotency in *Ezh2*-knockout mouse ESCs (Lavarone et al., 2019; Shen et al., 2008). Mechanistically, EZH1 is sufficient to preserve the H3K27me2/me3 at PcG target sites previously deposited by EZH2 and thus recruits PRC1 to further induce gene repression, although it fails to promote the propagation of H3K27me3 around the PRC2 sites (Lavarone et al., 2019). Compared with mouse ESCs, *EZH2*-knockout human ESCs show more compromised phenotypes associated with self-renewal and proliferation and increased transcription levels of mesodermal and endodermal genes (Collinson et al., 2016), suggesting that the compensatory role of EZH1 may differ under different contexts. During differentiation, EZH1 fails to rescue differentiation deficiency caused by the absence of EZH2 in both mouse ESCs and human ESCs (Shen et al., 2008). The deficiency of *Ezh2* does not affect early differentiation of human ESCs but mature differentiated cells will not be formed during the late stages, as revealed by the *in vitro* teratoma assay (Collinson et al., 2016). In mouse ESCs, embryoid bodies (EBs) of *Ezh2* knockout, *Ezh1/2* double-knockout and *Ezh2* mutant lacking enzymatic activity all show differentiation defects evidenced by lower expression of lineage markers (Lavarone et al., 2019). Such disruption of transcriptional program affects neuronal fate decision, based on data from a conditional *Ezh2*-knockout mouse model, possibly due to the remaining high levels of pluripotent genes (Buontempo et al., 2022). In addition, differentiation program depends on proper cell cycle status. It has been reported that when mouse ESCs exit G1 and enter G2, EZH2 accumulates at the promoters of lineage genes and enhances H3K27me3 signal, which may help to confine the cells to G1 and promote RNA synthesis (Asenjo et al., 2020). The H3K27 methyltransferase activity of EZH1 is generally considered as weaker than that of EZH2, but EZH1 can still take part in lineage differentiation. One recent report has shown that both loss-of-function and gain-of-function mutants of *EZH1* can affect human cortical neuronal differentiation but with different mechanisms: loss-of-function mutant maintains the proliferative properties of neural progenitor cells, whereas gain-of-function mutant promotes neuronal differentiation (Gracia-Diaz et al., 2023).

EZH2 traditionally acts as an epigenetic repressor in the PRC2 complex, but a growing number of evidence suggest that it could play a role in transcriptional activation (Gonzalez et al., 2011; Kim et al., 2018; Wang et al., 2022; Xu et al., 2012). For instance, in advanced prostate cancer, EZH2 participates in gene repression in an enzyme-activity-dependent manner, but also acts as a transcriptional activator to occupy promoters of androgen receptor genes (Kim et al., 2018; Xu et al., 2012). In acute leukaemias, the cryptic transactivation domain of EZH2 directly interacts with cMyc and other co-activators to promote oncogenesis (Wang et al., 2022). In breast cancer, EZH2 is reported to activate *AKT* isoform 1 and thus activate PI3K/AKT pathway (Gonzalez et al., 2011). However, whether dual regulation of EZH2 plays a role in stemness maintenance and early differentiation remains unclear and is worth exploring.

### 2.2 SUZ12: involves in the *de novo* establishment of H3K27me3

SUZ12, crucial for H3K27me3 establishment and EZH2-EED complex formation (Cao & Zhang, 2004), can contribute to early development through regulating H3K27me2/me3. SUZ12 binds to cell type-specific promoters, and most of the regions are almost identically occupied by the PRC2 complex and possess the repressive mark H3K27me3 in embryonic cells (Squazzo et al., 2006). In *Suz12*-knockout ESCs, EZH2 binding and H3K27me3 signal are strongly reduced (Højfeldt et al., 2018; Pasini et al., 2007), suggesting that SUZ12 might function upstream of EED and EZH2 and is the initial factor for *de novo* establishment of H3K27me3. Consistent with this notion, when H3K27me3 is severely impaired in mouse ESCs after the treatment of EZH2 inhibitor, SUZ12 remains to target CGIs; however, in *Suz12*-knockout cells, no EED and EZH2 binding along with H3K27me3 can be found though these regions belong to PRC2 target sites under normal condition (Højfeldt et al., 2018). Furthermore, the paternally imprinted *H19* is significantly upregulated in *Suz12*-knockout mouse ESCs, suggesting the role of SUZ12 in recruiting EED, which is important to propagate the H3K27me3 around the imprinted genes (Pasini et al., 2007). As the potential pioneer for mediating the PRC2 complex, SUZ12 is indispensable for both pluripotency maintenance and proper lineage differentiation. The expression profile of mouse ESCs upon *Suz12* knockout switches toward a differentiated state due to the high expressions of differentiation genes such as *Gata1*, *Gata4*, and *Hnf4* as the consequence of H3K27me3 loss. EBs derived from *Suz12*-knockout mouse ESCs exhibit differentiation defects and fail to form mature neurons upon RA induction (Pasini et al., 2007). Mouse embryos lacking *Suz12* show a loss of H3K27me2/me3 and the post-implantation development is severely impaired (Pasini et al., 2004). All these phenotypes support that SUZ12 is required for early differentiation.

Strikingly, fusion of SUZ12 and JAZF occurs frequently in endometrial stromal sarcoma and affects differentiation and oncogenesis (Koontz et al., 2001; Li et al., 2007). Stably expressing the fusion form of JAZF1-SUZ12 in ESCs leads to abnormal activation of PRC2 target genes that are supposed to be suppressed during EB differentiation, whereas JAZF1-bound sites are abnormally inhibited (Tavares et al., 2022), suggesting that the JAZF1-SUZ12 regulates cell differentiation. Mechanistically, SUZ12 is absent of N-terminus in the fusion protein, and thus loses the ability to bind to other PRC2 accessory proteins, resulting in less PRC2 occupancy and lower H3K27me3 enrichment at PRC2 target sites; however, the fusion protein confers the ability to target SUZ12 to the JAZF1-binding site, which is then occupied by the PRC2 complex for gene repression (Tavares et al., 2022). SUZ12 is thus important for positioning PRC2, and its interactions with other factors including long noncoding RNAs and epigenetic enzymes often promote the recruitment of the PRC2 complex. We will discuss this issue in subsequent sections.

### 2.3 EED: contributes to the propagation of H3K27me3

The other core component EED can act as an epigenetic switcher in cellular identity transition as it can inhibit both pluripotency and differentiation-associated factors. Using nucleosome-binding assay, Margueron and colleagues reported the tight interaction between PRC2-EED and H3K27me3-deposited nucleosomes, and further investigation showed the carboxy-terminal domain of EED contributed to the propagation of the repressive mark (Margueron et al., 2009). Mouse ESCs without EED stay in an incomplete pluripotent state due to consistently abnormal expression of lineage-specific genes, as loss of *Eed* disrupts the H3K27me3 accumulation at the PRC2 target sites (Boyer et al., 2006). To balance the X-chromosome linked dose effect, mouse embryos undergo X chromosome inactivation during early development, which is mediated by the enrichment of PRC2 component EED on the X chromosome. Using *Eed*-knockout trophoblast stem cells, Kalantry and colleagues found EED inhibited gene reactivations and maintained transcriptional memory on the X chromosome during differentiation (Kalantry et al., 2006). In addition, EBs derived from *Eed*-knockout ESCs show similar gene expression patterns as undifferentiated ESCs, indicating that EED inactivation prevents differentiation state acquisition possibly by reducing H3K27me3 on pluripotent genes (Obier et al., 2015). The *Eed-*null mice show disrupted anterior-posterior patterning, demonstrating EED as a vital modulator to regulate embryogenesis (Schumacher et al., 1996). Moreover, EED promotes cortical neurogenesis by binding to the promoter of *Gli3*, a repressor of the Hedgehog signaling pathway. Deletion of *Eed* leads to the loss of H3K27me3 at *Gli3* promoter and aberrant accumulation of H3K27ac, which subsequently causes massive apoptosis and DNA damage in neural stem/progenitor cells (Zhang, Dai, et al., 2022). The EED-GATA6-P21 axis also precisely regulates neural stem/progenitor cell proliferation in subventricular zone during neurogenesis (Sun et al., 2018). Interestingly, using conditional *Eed-*knockout mice, Liu and colleagues found the ablation of *Eed* reduced the expression of SOX11 and further revealed the significant reduction of H3K27me1 at *Sox11* promoters (Liu et al., 2019). This report indicates that EED may participate in transcriptional activation in a H3K27me3-independent manner.

### 2.4 JARID2: fine-tunes the balance of H3K27me3

The core components of PRC2 lack DNA binding ability. JARID2 is a catalytically inactive histone demethylase with nucleosome and RNA binding capacity (Kaneko, Bonasio, et al., 2014; Son et al., 2013). Although being a non-core subunit in PRC2.2, it can recognize EED and PRC1-mediated histone H2A lysine 119 mono-ubiquitination (H2AK119ub), thus participates in the recruitment of PRC2 and regulates its methyltransferase activity (Kasinath et al., 2021; Peng et al., 2009; Shen et al., 2009). JARID2 orchestrates the balance between pluripotency maintenance and lineage commitment through fine-tuning H3K27me3. *Jarid2*-knockout mouse ESCs possess normal colony phenotype although an earlier and more pronounced downregulation of pluripotency factors occurs. Mechanistically, it may be due to the redistribution of H3K27me3 across the genome as SUZ12, EZH2 and EED bindings are remarkably reduced in the absence of *Jarid2* (Sanulli et al., 2015). During lineage differentiation, *Jarid2*-knockout ESCs exhibit the dysregulated expression pattern of neural and mesendodermal genes over the course (Landeira et al., 2010; Shen et al., 2009). *Jarid2* deficiency could cause reduced enrichment of RNAP at the target sites, which is supposed to occupy at the PRC2 target, poised to prime transcription (Landeira et al., 2010), suggesting a possible interaction between RNAP and JARID2 as well as the importance of JARID2 in safeguarding the poised state of RNAP. In naïve mouse ESCs, the FGF/ERK signaling protein could recruit JARID2 to the bivalent FGF signaling target genes and influence the enrichment of PRC2, as an effective way to increase transcriptional thresholds of developmental genes (Aljazi et al., 2020) (Fig. 2). On the other hand, the cell signaling can rapidly elicit bivalent gene expression via its own transcriptional activation ability at target sites (Aljazi et al., 2020). Contradictory views exist on whether JARID2 promotes or inhibits PRC2 trimethyltransferase activity (Landeira et al., 2010; Shen et al., 2009). Moreover, H3K27me1 and H3K27me2 change dramatically in the *Jarid2*-knockout ESCs, raising important questions including how JARID2 modulates these two modifications and what the consequence is for transcription regulation.

Since PRC2 plays a crucial role in establishing H3K27me3, there are close associations between PRC2 mutations and developmental disorders or diseases. *Eed* and *Ezh2* deletion led to aberrant gastrula development in mice (O'Carroll et al., 2001) and digestive organ deficiencies in zebrafish (Raby et al., 2021). *EZH1* loss-of-function variants such as frame shift mutation or deletion mutation, and *EZH1* gain-of function variants including certain missense mutations could cause neurodevelopmental disorders in human (Gracia-Diaz et al., 2023). In addition, many reports have reported that the dysregulation or mutations of *EZH2* are closely related to the progression of solid tumors and hematologic tumors. In breast cancer, abnormal expression of EZH2 promotes the growth and invasive ability of cancer cells (Kleer et al., 2003). The high expression of EZH2 is involved in the progression of prostate cancer (Ren et al., 2012; Varambally et al., 2002). Supporting with those observations, somatic mutation of Y641 in EZH2, which alters the substrate preferences and functions as a gain-of-function variant, accelerates the proliferation of diffuse large B-cell lymphoma (McCabe et al., 2012). However, the activation of NOTCH1 signal results in reduced H3K27me3 level by genetic inactivation of PRC2, and thus promotes the malignancy in T-cell acute lymphoblastic leukemia, which underlines the importance of EZH2/PRC2 to inhibit the oncogenesis (Ntziachristos et al., 2012). The contrasting effects of EZH2 in different tumor types demonstrate the complexity of its regulation. Of note, due to the importance of its function, targeting EZH2 has been applied in *EZH2* activation mutant lymphoma and diffuse intrinsic pontine glioma (McCabe et al., 2012; Mohammad et al., 2017).

Of note, the exclusive subunits involved in the PRC2.1 or PRC2.2 may have different roles in cell identity transition. Recent reports have shown that both JARID2 and MTF2 (also known as PCL2) can safeguard proper differentiation to various extents. Upon spontaneous differentiation of EBs, *Mtf2*-knockout cells display a strong tendency to differentiate in concomitant with high expression of marker genes in mesendoderm and low expression of pluripotent genes, but *Jarid2*-knockout EBs have much milder phenotypes although still tend to favor mesendoderm differentiation (Loh et al., 2021). In addition, the exclusive subunits of the two PRC2 subtypes could restrict mouse ESCs to the G1 phase in the cell cycle through different mechanisms: JARID2 and EZH2 of PRC2.2 co-bind to the bivalent promoters including *Hoxd* gene cluster during S and G2-M phases, and EPOP of PRC2.1 acts as a transcriptional activator that binds to the bivalent genes in G1 phase and maintains the cells in G1 phase (Asenjo et al., 2020).

In summary, PRC2 can function not only directly with enzymatic activity but also through maintaining a topological structure between gene regulatory elements and target genes (Fig. 2). Poised enhancers are a type of regulatory elements that are bound by transcription factors and coactivators (Rada-Iglesias et al., 2011), and the flanking nucleosomes of them are modified by H3K27me3. In mouse ESCs, the poised enhancers regulate the activation of major anterior neural genes by forming a chromosome loop structure during differentiation, and the PRC2 complex participates in such process and promotes the activation of poised enhancers to initiate differentiation (Cruz-Molina et al., 2017).

## 3 The removal and recognition of H3K27me3

In the pluripotent state, the H3K27me3 transcriptional repression landscape established by PRC2 on lineage-specific genes is important for stemness maintenance, whereas the removal of H3K27me3 plays a vital role for cell identity transition under appropriate differentiation conditions, along with the establishment of active mark H3K4me3. The KDM6 subfamily is the class responsible for H3K27 demethylation, which consists of KDM6A (also named UTX), KDM6B (also named JMJD3), and KDM6C (also named UTY) (Fig. 1) (Manni et al., 2022). Among them, KDM6A and KDM6B are the most common H3K27 demethylases of mammals and participate in H3K27me2/me3 demethylation. KDM6A and KDM6B promote the reduction of H3K27me3 on *HOX* genes during differentiation and are important for development (Agger et al., 2007). Depletion of *KDM6A* and *KDM6B* in human ESCs significantly enhances early neuroectoderm differentiation and impairs definitive endoderm differentiation, likely via activating the WNT genes in a demethylation-dependent manner (Jiang et al., 2013; Meng et al., 2021). At the later stages of differentiation, deletion of *KDM6A* and *KDM6B* in human neural progenitor cells results in significant apoptosis and failure of neuronal differentiation (Shan et al., 2020). The abnormal accumulation of H3K27me3 at the neuron-specific genes caused by *KDM6* deletion thus reduces chromatin accessibility assayed by ATAC-seq (Shan et al., 2020). Another study suggests that KDM6A could demethylate the H3K27me3 at the *Pten* promoter, thus inhibiting the proliferation of neural progenitors and promoting neuronal differentiation (Lei & Jiao, 2018). Tang and colleagues reported that the loss of *KDM6A* in neurons resulted in the downregulation of neural developmental markers; further assessment revealed the regions, which should have reduced H3K27me3 levels, still showed H3K27me3 enrichment (Tang et al., 2020). These reports together indicate that KDM6 affects neural lineage differentiation by participating in the regulation of either chromatin accessibility or signaling pathways of neural-related markers via H3K27 demethylation. Interestingly, KDM6A is highly expressed in many cell types, whereas KDM6B is highly expressed only under developmental stimulations. The distinctive expression patterns among the two enzymes suggest they may exert demethylation in different manners (Chen et al., 2012). KDM6B appears to be associated with the transcription elongation process. KDM6B can reduce H3K27me3 level at the bivalent regions and further combine with the elongation factors including SPT6 and SETD2 to promote continued elongation of RNA polymerase II (Chen et al., 2012). In RA-induced differentiation of mouse ESCs, the loss of *Kdm6a* reduced the expression of some RA-activated developmental genes such as *Notch1*, *Pax6* and *Pax3*, supporting the critical role of KDM6A in bivalency resolution (Dhar et al., 2016). In addition, KDM6A promotes the expression of neuronal differentiation-associated genes by increasing H3K27ac with little impact on H3K27me3 and suppresses AP-1 thus limiting gliogenesis (Xu et al., 2020).

Vitamin C is an important cofactor for KDM6 activity. In the absence of Vitamin C, hematopoietic stem cells fail to undergo endothelial-to-hematopoietic transition because low KDM6 activity leads to aberrant accumulation of H3K27me3, which prevents chromatin opening at those gene loci that promote hematogenesis (Zhang et al., 2019). The cooperation of Vitamin C and KDM6A changes the epigenetic states of metabolic and pluripotent associated genes and increases the reprogramming efficiency of iPSCs (Esteban et al., 2010; Jiang et al., 2020; Mansour et al., 2012).

Bromo-Adjacent Homology (BAH) domain harbored proteins are major interpreters for H3K27me3, which finely safeguard the repressive mark at target genes. Some reports have revealed that the BAH domain recognizes H3K27me3 in Arabidopsis Thaliana, fungi, and mammals (Qian et al., 2021; Tang et al., 2021; Zhang et al., 2020). BAHCC1 is a BAH-domain containing module highly expressed in human acute leukemias. The disruption of *BAHCC1* leads to abnormal de-repression of target genes in JURKAT cells and promotes oncogenesis (Fan et al., 2020). In HEK293T cells, the point mutation of *BAHD1* causes the increased histone acetylation at polycomb repressive targets and the knockout of *Bahd1* results in abnormal placental and fetal growth in mice (Fan et al., 2021). Of note, PRC1 can recognize H3K27me3 and further contribute to the chromatin compaction specifically via CBX2/4/6/7/8 (van Wijnen et al., 2021).

## 4 Additional factors that influence H3K27me3

**Fig. 3:**
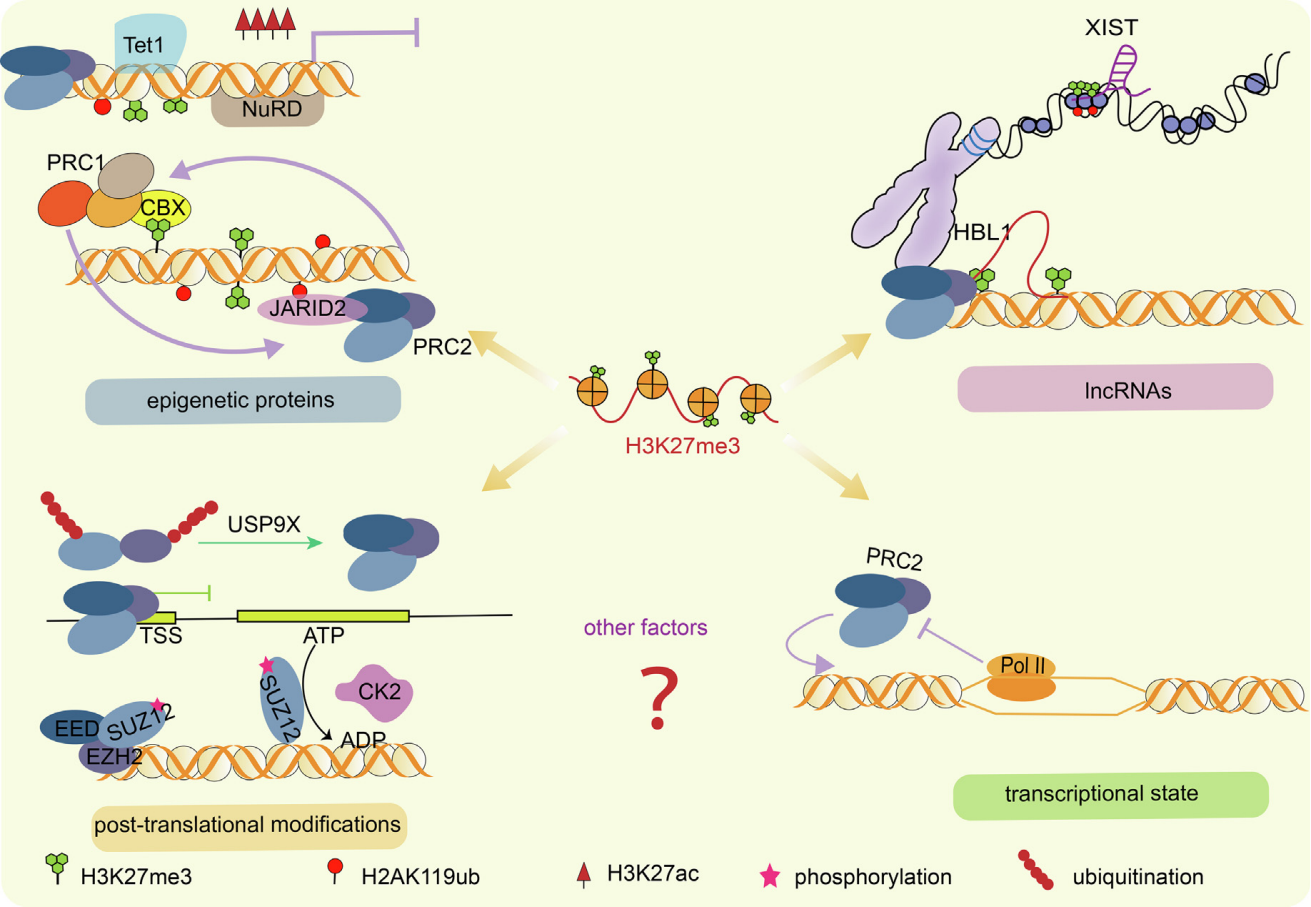
**The important factors that influence H3K27me3.** Other epigenetic factors, such as TET1 protein, PRC1 and NuRD complex, can crosstalk with PRC2 and modulate H3K27me3; lncRNAs binds to PRC2 and recruits the complex to specific genome regions; post-translational modifications on PRC2 components can alter the activity or recruitment; transcriptional state on local regions also affect the PRC2 action.

The occupancy of H3K27me3 is tightly correlated with the assembly and recruitment of the PRC2 complex, as well as the stability of each subunit. Many other factors have been reported to influence H3K27me3, including proteins such as non-core subunits of PRC2, other epigenetic enzymes and transcriptional factors, and the other PcG protein complex PRC1, as well as RNAs including long noncoding RNAs (lncRNAs). In addition, the transcriptional state also affects PRC2 binding (Fig. 3).

As two repressive complexes of PcG proteins, PRC1 and PRC2 often synergistically inhibit the transcription. PRC1 can deposit H2AK119ub to facilitate condensing the chromatin. It's widely accepted that PRC2 and PRC1 can communicate and cooperate with each other through a positive feedback mechanism (Fursova et al., 2019). H3K27me3 deposited by PRC2 can direct PRC1 binding at the target genes, and this is achieved by CBX (chromobox) protein of PRC1, which can recognize H3K27me3 (Francis et al., 2004).

JARID2 and AEBP2 of PRC2.2 can recognize the H2AK119ub and further strengthen the feedback between PRC1 and PRC2 through promoting the H2AK119ub-dependent H3K27me3 modification (Højfeldt et al., 2019). The two complexes synergistically repress gene transcription (Blackledge & Klose, 2021; Laugesen et al., 2019). Moreover, the methylation at JARID2 itself (K116me3) by PRC2 can reciprocally promote allosteric activation of PRC2 enzymatic activity (Kasinath et al., 2021; Sanulli et al., 2015). Of note, PRC2 exists in two subtypes PRC2.1 and PRC2.2 that can function separately and collaboratively, and the chromatin localization of PRC2.1 and PRC2.2 depends on PRC1 to certain extents. After the loss of H2AK119ub by *RingA/B* knockout, JARID2 undergoes a substantial displacement on chromatin, whereas no change in the chromatin binding level of MTF2 occurs. It's likely that PRC1 affects JARID2-PRC2.2 localization and further induces H3K27me3 change on the polycomb region (Healy et al., 2019). In mouse ESCs carrying conditional point mutant causing PRC1 enzymatic inactivation, the reduction of SUZ12 and H3K27me3 at the target regions occurs, suggesting that PRC1 enzymatic inactivation affects PRC2 localization and H3K27me3 deposition (Blackledge et al., 2020). This is consistent with other studies showing an important role of PRC1 in the recruitment of PRC2, the accumulation of H3K27me3, and the polycomb-related gene repression (Blackledge et al., 2014; Cooper et al., 2014).

In addition, the DNA dioxygenase TET1 can recruit EZH2 and influence the H3K27me3 deposition on the developmental bivalent genes independent of its enzyme catalytic activity. The *Tet1*-knockout, but not *Tet1* enzymatic mutant mouse ESCs, experience abnormal upregulation of mesendoderm and trophectoderm markers, and the EB differentiation assay also reveals robust expression of *Tet1* in all three germ layer markers. Mechanistically, nearly 40% of TET1 target regions are overlapped with EZH2 target sites and loss of *Tet1* diminishes the H3K27me3 deposition on these regions (Chrysanthou et al., 2022; Huang et al., 2022). Whether the other two homologs, TET2 and TET3, are also involved in interactions with PRC2 components and play a role in different cell types are worthy of exploring. As a repressive complex through de-acetylating H3K27, NuRD targets most of the bivalent promoters, which are occupied by H3K4me3 and H3K27me3 and are generally enriched for PRC2. Upon NuRD depletion, the binding of PRC2 at target genes is reduced revealed by SUZ12 ChIP-seq, supporting that the two repressive complexes cooperate to regulate lineage commitment by deacetylating and tri-methylating H3K27 (Reynolds et al., 2012).

Protein post-translational modifications can affect H3K27 regulation as well. Through reducing the ubiquitination of SUZ12 and EZH2, the deubiquitinating enzyme USP9X stabilizes the PRC2 complex. Upon *Usp9x* depletion, the level of H3K27me3 is significantly decreased on those developmental genes which are normally inhibited by H3K27me3 (Macrae & Ramalho-Santos, 2021). In addition to ubiquitination, phosphorylation of SUZ12 is also significant in the regulation of PRC2 complex. Casein kinase 2 (CK2) can phosphorylate S583 residue of SUZ12 to stabilize the methyltransferase activity of PRC2 and promote SAM recruitment, a critical metabolite to histone methylation. The phosphorylation of SUZ12 mediated by CK2 is also important to exit from pluripotency and maintain differentiated cell identity as the mouse EBs carrying SUZ12^S583A^ which loses the phosphorylation could revert to pluripotent state in 2i medium (Gong et al., 2022).

RNA seems necessary for H3K27 methylation and PCR2 function. Using RNase-dependent ChIP (r-ChIP), Long and colleagues found the inhibition of nascent RNAs disrupted the H3K27me3 localization at target loci but increased the occupancy at non-target loci (Long et al., 2020). They further constructed mutant human PSCs with RNA-binding deficient PRC2 and performed cardiac differentiation, which showed that PRC2 required RNAs for its role in cell fate regulation (Long et al., 2020). Another study also found the human-specific heart-brake lncRNA 1 (*HBL1*) was required for PRC2 occupancy on cardiac genes. In *HBL1*-null human iPSCs, EED and H3K27me3 binding were notably reduced on mesoderm and heart developmental genes (Liu et al., 2021). Very recently, lncRNA *XIST* which regulates the X chromosome inactivation, was reported to involve in recruiting PRC2 and establishing H3K27me3 in order to dampen the autosomal X chromosome-linked genes expression (Dror et al., 2024). In addition, there are some studies suggesting that RNA inhibits PRC2 and nucleosome interactions (Beltran et al., 2016; Kaneko, Son, et al., 2014). It is worth thinking about how exactly these RNAs participate in PRC2 recruitment and the subsequent functional modulation: do they rely on their specific structures to enhance the interactions with PRC2 through RNA complexes, or do they confer site-binding specificity for PRC2 through base-pairing with DNA? We should also mention that there are controversies for RNA-based regulations of PRC2, particularly considering that the interactions between RNAs and PRC2 complex are not very specific (Kretz & Meister, 2014; Zhao et al., 2010). Recently, the cryo-electron structures showed that G-quadruplex RNA could promote PRC2 dimerization and interfere with the interactions between PRC2 and nucleosomes (Song et al., 2023). This observation further supports that the interactions between RNAs and PRC2 largely depend on RNA structure and specific characteristic of certain RNA motif. However, another work utilized denaturing purification of *in vivo* crosslinked RNA-protein complexes and concluded that the RNA-PRC2 interactions may not occur (Guo et al., 2024). Nevertheless, whether there is any or to what extent the RNA dependency of PRC2 exists needs further clarifications.

It's intriguing that transcriptional status seems tightly associated with PRC2 recruitment. By blocking the transcription through chemicals, Riising and colleagues detected more SUZ12 peaks and more enrichment of H3K27me3 in a genome-wide scale. Some of the PRC2 targets, such as *Fgf4* and *Utf1*, obtained SUZ12 binding and H3K27me3 modifications in mouse ESCs, although they are normally deposited by H3K27me3 only during differentiation (Riising et al., 2014). Kaneko and colleagues used the transcription repressor polyadenylation signal to silence the CGIs-associated promoters and found the same result (Kaneko, Son, et al., 2014). These results were found in the pluripotent state, whether the use of transcription repressors or activators would cause an abnormal distribution of PRC2 when cells are differentiated is also worthy of deeper investigation. Of note, most transcription factors associated with self-renewal and pluripotency are co-occupied on the PRC2-bound silencers, and thus they may play a supporting role for the transcription repression function of PRC2 (Ngan et al., 2020). In addition, co-localization of H3K27me3, EZH2 and transcription factor BATCH1 is found across the genome, and the absence of *BATCH1* causes reductions of H3K27me3 and EZH2 binding on the mesendodermal genes, indicating BATCH1 impedes mesendoderm differentiation by recruiting PRC2 (Wei et al., 2019). MEN1, which functions in the regulation of histone modification and epigenetic regulation, can regulate H3K27me3 levels at the bivalent genes in human ESCs, and the inhibitor of MEN1 can dramatically depress the PRC2 target genes such as *CXCR4* and *CD13* (Sparbier et al., 2023). BEND3 is a sequence-specific DNA binding transcriptional repressor which highly enriches at bivalent promoters of developmental genes (Kurniawan et al., 2022), and the loss of *Bend3* causes reduced H3K27me3 in mouse ESCs, suggesting the essential role of BEND3 for recruiting PRC2 and preventing the premature activation of developmental genes (Zhang, Zhang, et al., 2022). Recently, Matsui and colleagues found the pioneer factor FOXA cooperates with PRDM1 to recruit PRC1, which further promotes H3K27me3 deposition and the establishment of bivalent domains of alternative lineage-enhancers in human definitive endoderm (Matsui et al., 2024).

Strikingly, several studies have reported the crosstalk between H3K27me3 and other histone marks, suggesting the intricate regulatory mechanism mediated by epigenetic modifications. For instance, H3K36me3 inhibits the PRC2 activity and antagonizes H3K27me3 (Schmitges et al., 2011; Yuan et al., 2011). Mechanistically, the H3K36me3 writer SET2 methylates EZH2 and inhibits its activity. In *Set2*-deficient mice, high expression of EZH2 and increased H3K27me3 level are observed. On the contrary, the *Set2* mutation impairs the interaction between the two core enzymes, suggesting the antagonistic effect (Yuan et al., 2020). It has also been shown that H3K27me3 can antagonize H3K18ub and DNA methylation. Mechanistically, H3K27me3 inhibits UHRF-mediated ubiquitination of H3 and further affects DNA methylation by DNMT1 (Zhang, Liu, et al., 2022). More interestingly, similar to H3K27me3, another inhibitory mark H3K9me3, exhibits co-localization with H3K4me3 in certain genes to maintain those target genes in a poised state, suggesting that the two repressive hallmarks may cooperatively regulate cell fate determination (Torres-Campana et al., 2022). Taken together, the communications among histone modifications provide a huge space for the precise regulation of gene expression.

To summarize, PRC2 interacts with other factors (Fig. 3), which might provide specificity and diversity, and finely safeguard cell fate and orchestrate a multiplicity of accurate differentiation programs.

## 5 Conclusion and perspectives

H3K27me3 is the critical epigenetic repressive hallmark. So far, we have summarized and discussed how the repressive mark H3K27me3 and the writer PRC2 as well as the eraser KDM6 influence pluripotency and early differentiation. In the pluripotent state, PRC2 safeguards cell identity by inhibiting the expression of developmental genes. During lineage differentiation, accurate identity transition is accompanied by suppressing non-lineage genes and derepressing the lineage genes orchestrated by PRC2 and KDM6. Of note, the co-occupation of H3K4me1 and H3K27me3 is an important feature of the primed enhancer, which could be an aspect of cell type specific transcriptional regulation. The H3K4me1-H3K27me3 transition can regulate tissue-specific gene expression. In mouse ESCs, *Eed* or *Suz12* deficiency directly induces the H3K27me3-H3K4me1 transition, leading to up-regulation of mesodermal markers and down-regulation of ectodermal markers (Yu et al., 2023). In non-small cell lung cancer, the loss of *KDM6A* increases the level of H3K27me3 on the enhancers of neuroendocrine cells and decreases the enrichment of H3K4me1, and thus leading to the transformation of the ASCL1 cell subtype of non-small cell lung cancer to the NEUROD1 cell subtype (Duplaquet et al., 2023). The maintenance of the primed state by the two modifications is an important way to achieve rapid cell identity transition.

Importantly, the repressive mark H3K27me3 seems to be a barrier in the cell fate transition. In addition to modulating the balance between pluripotency and differentiation, H3K27me3 also safeguards naive human PSCs from trophectoderm and mesoderm differentiation, as well as controls the transition between naive and primed states (Kumar et al., 2022). Then how does PRC2 achieve rapid and precise regulation? Transcription factors and lncRNAs may be the keys. Transcriptional factors offer the opportunity for PRC2 complex to rapidly anchor to target gene loci due to their DNA-binding specificities (Kurniawan et al., 2022; Yeh et al., 2023), and lncRNAs could guide PRC2 to the appropriate loci through being complementary to DNA. It is noteworthy that contradictory views exists on whether and how RNAs interact with PRC2, as rChIP-based experimental methods may have inherent deficiencies (Hickman & Jenner, 2024).

Chemical-based regulation of H3K27me3 and PRC2 function is widely applied in various biological contexts. For instance, chemical reprogramming offers promising applications for modulating cell fate and generating pluripotent stem cells (Wang et al., 2023). The EZH2 inhibitor DZNep has been widely used in reprogramming of mouse PSCs and human PSCs (Guan et al., 2022; Zhao et al., 2015), and the *EZH1*-knockdown iPSCs-derived T cells exhibit potent antitumor ability (Jing et al., 2022), together underlying the importance of epigenetic regulation of PRC2 in cell fate conversion and cell therapy. In addition, given that SAM is the catalytic substrate for H3K27 methylation and Vitamin C as well as α-KG are the cofactor for KDM6, these metabolites are powerful modulators for H3K27me3 as well, despite other demethylases are also affected in certain extent. Taken together, as the H3K27me3 pattern finely regulates the balance between pluripotency and lineage differentiation, H3K27me3-based methods may offer widely functional applications in the near future.

## CRediT authorship contribution statement

**Liwen Jiang:** Writing – original draft, Visualization, Methodology, Investigation, Formal analysis. **Linfeng Huang:** Writing – review & editing, Supervision. **Wei Jiang:** Writing – review & editing, Supervision, Funding acquisition, Conceptualization.

## Declaration of competing interest

The authors declare that they have no known competing financial interests or personal relationships that could have appeared to influence the work reported in this paper.
